# Comparison of Detection of Superior Gluteal Artery Perforator by Indocyanine Green Fluorescence Near-Infrared ANGIOGRAPHY and Handheld Acoustic Doppler Sonography for Reconstruction of Sacral Pressure Injury

**DOI:** 10.3390/jpm12020132

**Published:** 2022-01-19

**Authors:** Chien-Wei Wu, Hung-Hui Liu, Chun-Yu Chen, Kuo-Feng Hsu, Yu-Yu Chou, Dun-Wei Huang, Yuan-Sheng Tzeng

**Affiliations:** Division of Plastic and Reconstruction Surgery, Department of Surgery, Tri-Service General Hospital, National Defense Medical Center, Taipei 114, Taiwan; alex199180220@hotmail.com (C.-W.W.); liu0934303739@gmail.com (H.-H.L.); illusioncjy@gmail.com (C.-Y.C.); captain0416@gmail.com (K.-F.H.); justyu1112@gmail.com (Y.-Y.C.); gdream12731@gmail.com (D.-W.H.)

**Keywords:** handheld acoustic Doppler sonography, indocyanine green fluorescence near-infrared angiography, sacral pressure injury, superior gluteal artery perforator

## Abstract

Aims: Pressure injury is a gradually increasing disease in the aging society. The reconstruction of a pressure ulcer requires a patient and surgical technique. The patients were exposed to the radiation risk under other ways of detection of perforators such as computed tomographic angiography and magnetic resonance angiography. Here, we compared two radiation-free methods of a superior gluteal artery perforator (SGAP), flap harvesting and anchoring. One is the traditional method of detecting only handheld acoustic Doppler sonography (ADS) (Group 1). The other involves the assistance of intraoperative indocyanine green fluorescent near-infrared angiography (ICGFA) and handheld ADS (Group 2). Materials and Methods: This is a single-center, retrospective, observational study that included patients with sacral pressure injury grades III and IV, who had undergone reconstructive surgery with an SGAP flap between January 2019 and January 2021. Two detection methods were used intraoperatively. The main outcome measures included the operative time, estimated blood loss, major perforator detection numbers, wound condition, and incidence of complications. Results: Sixteen patients underwent an SGAP flap reconstruction. All patients were diagnosed with grade III to IV sacral pressure injury after a series of examinations. Group 1 included 8 patients with a mean operative time of 91 min, and the mean estimated blood loss was 50 mL. The mean number of perforators was 4. Postoperative complications included one wound infection in one case and wound edge dehiscence in one case. No mortality was associated with this procedure. The mean total hospital stay was 16 days. Group 2 included 8 patients with a mean operative time of 107.5 min, and the mean estimated blood loss was 50 mL. The mean number of perforators was 5. Postoperative complications included one wound infection. No mortality was associated with this procedure. The mean total hospital stay was 13 days. Conclusions: The combination of detection of the SGAP by ICGFA and handheld ADS for the reconstruction of a sacral pressure injury provides a more accurate method and provides the advantage of being radiation-free.

## 1. Introduction

Pressure injuries are areas with tissue necrosis and ulceration where soft tissues are compressed between hard surfaces and a bony structure. They are caused by mechanical pressure in combination with friction, shearing forces, and moisture. They can be a severe problem not only for patients and their caregivers, but also for plastic surgeons. Once deep tissue infection is noted, in-hospital care, along with surgical interventions, is beneficial for patients. Several different kinds of flaps have been introduced over the years to reconstruct sacral defects, such as local flaps, V-Y advancement fasciocutaneous flaps, gluteus maximum muscle-based flaps, inferior gluteal artery perforator flaps, and superior gluteal artery perforator (SGAP) flaps [1]. Reliable perforators are the most important factors for a successful perforator-based flaps reconstruction [2]. Thus, an accurate preoperative mapping of the perforators is essential for the safe planning of propeller flaps. To date, various methods have been reported: (1) handheld acoustic Doppler sonography (ADS), (2) color duplex sonography, (3) perforator computed tomographic angiography (P-CTA), (4) magnetic resonance angiography, and (5) indocyanine green fluorescence near-infrared angiography (ICGFA) [3,4]. Out of these ways of mapping perforators, the golden standards are perforator computed tomographic angiography (P-CTA) and the magnetic resonance angiograph. However, they are expensive for healthcare organizations. The most commonly used one during operations is the handheld acoustic Doppler sonography (by the operators). In recent decades, the new mapping technique of indocyanine green fluorescence near-infrared angiography (ICG drug was injected intravenously through the central or peripheral routes. A real-time laser fluorescent angiography was performed, and the points at which the ICG uptake were highest were observed) has provided us with a real-time and radiation-free method during operations.

Both ADS and ICGFA could be used during the SGAP operation. To the best of our knowledge, there is no study that compares these methods in the SGAP operation. The aim of this study was to compare the accuracy and feasibility of ADS with ICGFA and ADS only in the reconstruction of sacral pressure ulcers with an SGAP flap.

## 2. Materials and Methods

We retrospectively reviewed data from 16 patients (Table 1 and Table 2) with sacral pressure injury who underwent modified SGAP flap reconstruction surgery. All patients aged 65 years and over with a sacral pressure injury stage III to stage IV and with a massive soft tissue defect coverage with an SGAP flap between January 2019 and January 2021 who met the inclusion criteria were included in this study.

This study protocol was reviewed and approved by the Institutional Review Board of the Tri-Service General Hospital (TSGHIRB No.:C202105029). It was conducted in compliance with the Helsinki Declaration.

We compared two methods for the detection of SGAP flap harvesting and anchoring. One is the traditional method of detecting only handheld ADS (Group 1). The other method involves the assistance of intraoperative ICGFA and handheld ADS (Group 2). Patients with a history involving a failing microvascular flap, hyperthyroidism, a known hypersensitivity toward indocyanine green (ICG) or related substances, as well as patients currently participating in another clinical study, were excluded from this study. The enrolled patients were provided with coherent information about the study and possible complications associated with ICG application, and they provided written informed consent. The body mass index, American Society of Anesthesiologists status, mobility status (such as diabetes mellitus), nutritional status, and patient’s history regarding smoking and alcohol use were registered alongside the gender and age in order to analyze their potential role in a correct perforator localization. The perforator number, surgical time, flap size, outcome, and complications were recorded. Minor complications included partial dehiscence and necrosis. Major complications included flap failure and dehiscence. All statistical analyses were performed with IBM SPSS statistical software version 22 for MAC (IBM Corp., Armonk, NY, USA).

### Surgical Technique

**Figure 1:**
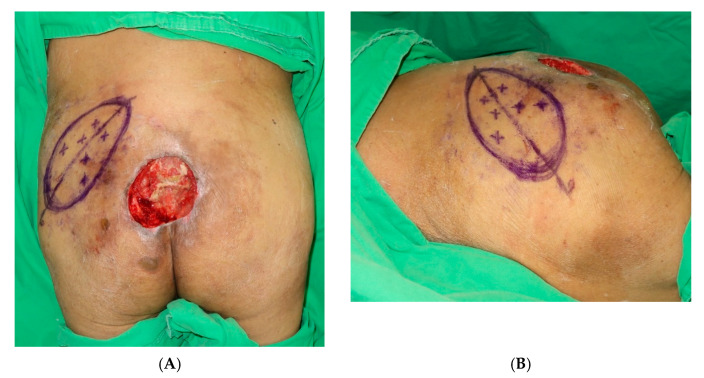
(**A**) A hypothetical line was drawn between the posterior superior iliac spine (PSIS) and the lateral border of the great trochanter on the side of the buttock from which the SGAP flap was planned to be harvested. (**B**) A hypothetical line was drawn between the posterior superior iliac spine (PSIS) and the lateral border of the great trochanter on the side of the buttock from which the SGAP flap was be harvested.

**Figure 2:**
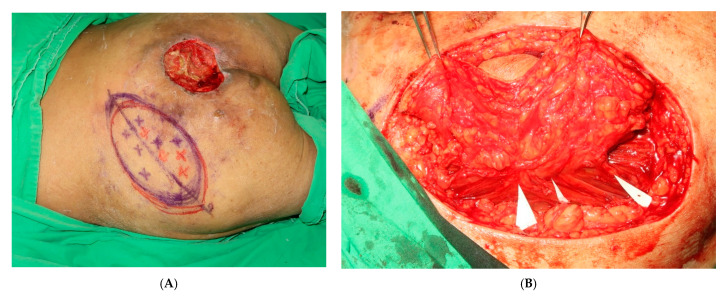
(**A**) The superior gluteal artery (SGA) and its perforators were identified with ICGFA and ADS. (**B**) The purple marks represent perforators detected by ICGFA; The red marks represent perforators detected by ADS.

The patients were prepared while they were in the prone position, under general anesthesia, in a warm operating room. After adequate debridement of the lesion, a hypothetical line was drawn between the posterior superior iliac spine and the lateral border of the great trochanter on the side of the buttock from which the SGAP flap was to be harvested (Figure 1A,B). After the anatomical landmarks were drawn, the superior gluteal artery (SGA) and its perforators were identified using ICGFA and ADS (Figure 2A,B).

In group 1, ADS was used for the detection of the perforators only, and in group 2, after ADS detection, a 0.1-mg/kg dose of ICG was planned to be injected intravenously through the central or peripheral routes. At the same time, real-time laser fluorescent angiography was performed using the SPY^®^ intraoperative perfusion assessment system (Novadaq Technologies Inc., Richmond, BC, Canada), and the points at which the ICG uptake were highest were observed. We marked both perforators that were detected by ICG and ADS. The skin paddle was designed using the ICG. We aimed to include the brightest perforators in the middle of the flap.

**Figure 3:**
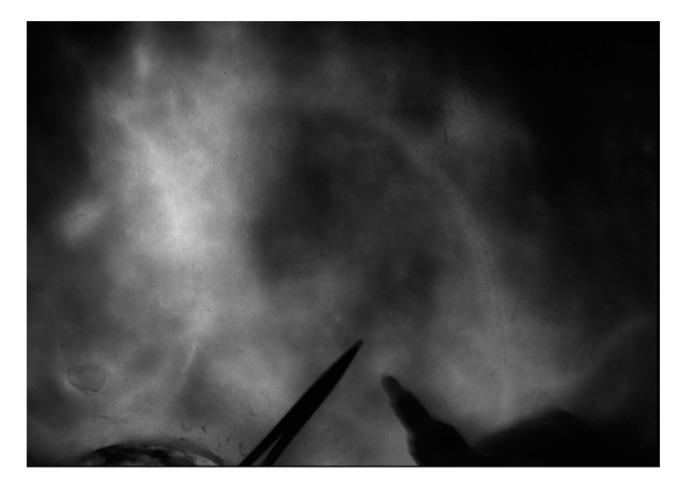
The perforators were identified.

**Figure 4:**
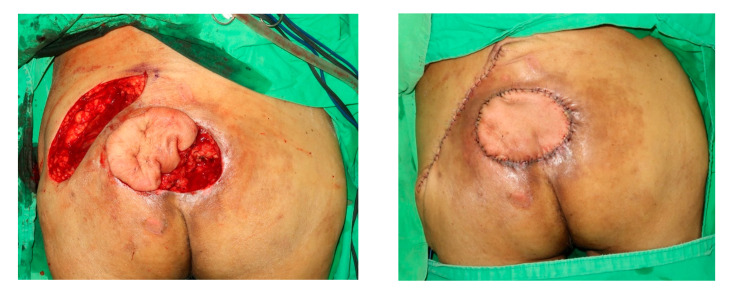
The SGAP flap was tunneled beneath a skin strip between the defect and the flap donor site.

The SGA perforators are situated mainly around the junction of the middle and medial third of the line drawn between the posterior superior iliac spine and the greater trochanter (Figure 3). A template of the defect was drawn on a sterile, exposed radiograph, which helped ensure the accurate size and shape of the recipient site and donor tissue. The flap template was placed on the perforator. To cover a smaller sacral sore (usually <8 cm in diameter), the SGAP flap was tunneled beneath the skin strip between the defect and the flap donor site (Figure 4). Additionally, to cover a larger sacral sore (>8 cm in diameter), the SGAP flap was designed to closely fit the defect. The defect was covered with a rotational SGAP flap [5].

## 3. Results

In group 1 (ADS-only) (Table 3), a total of eight patients were enrolled. Six flaps had one perforator, and two flaps had two perforators. The mean operative time was 91 ± 19 min. All flaps survived. Two patients developed minor complications, with partial necrosis. The mean total hospital stay was 16 days.

In group 2 (ADS and ICGFA) (Table 4), a total of eight patients were enrolled. Seven patients had two perforators detected by ICGFA, and one patient had three perforators detected by ICGFA. Compared to the ADS, the ICGFA could detect more reliable perforators. The mean operative time was 107.5 ± 15.7 min. All flaps survived. One patient developed minor complications, with partial necrosis. The mean total hospital stay was 13 days.

The patient characteristics are summarized using the total number and mean ± standard deviation. All statistical analyses were performed with IBM SPSS statistical software version 22 for MAC (IBM Corp., Armonk, NY, USA). We compared both groups. There are no statistically significant differences of age, flap size, or grades of pressure injury. The results were analyzed using the Wilcoxon Signed Rank Test. The results showed that the number of perforators, surgical time, and post-surgical admission days were statistically not significant (*p* > 0.05).

## 4. Discussion

The preoperative examination allowed us to confirm the following items for perforator and vascular pedicles: the location of the perforators, the detection of a dominant perforator with a rich blood flow, the course of perforators, the length of the vascular pedicle, including the perforators, and the identification of anatomic variables. In addition, the preoperative identification of perforators using these methods offers many advantages when choosing the perforators [3]. The detection of the perforator is important.

**Figure 5:**
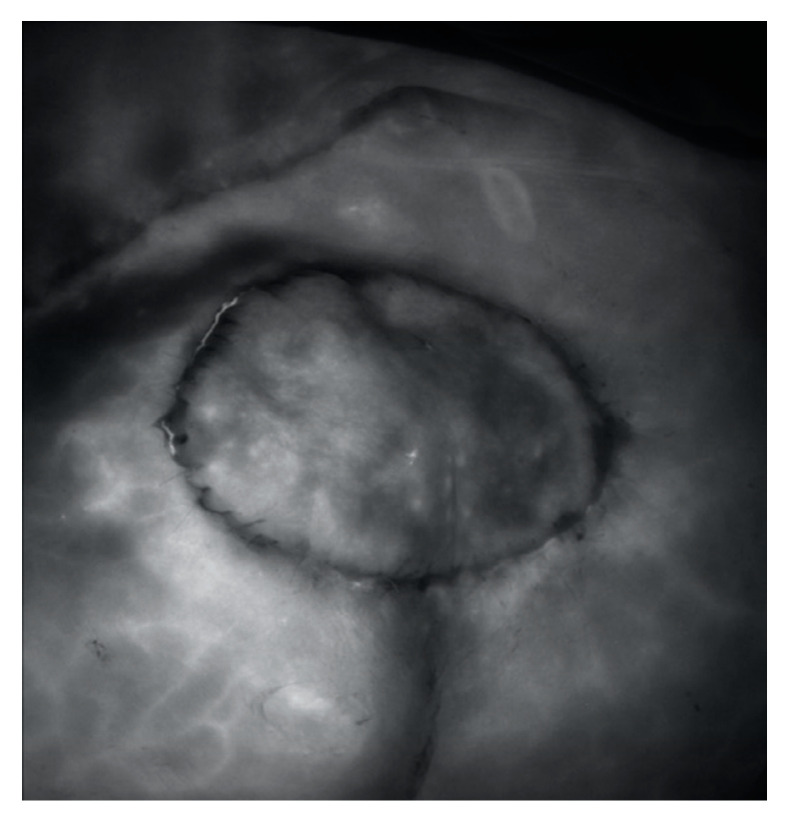
ICG fluorescent angiography was used to confirm a dynamic blood flow and provide a final confirmation of the perioperative posture of the perforators.

Traditionally, handheld ADS is an effective way of detecting the perforators of the SGAP flap (Figure 5). Plastic and reconstructive surgery has always been on the frontier of utilizing novel and innovative technologies to improve patient safety during operative procedures. ICG is a water-soluble tricarbocyanine dye that fluoresces upon exposure to near-infrared light. This fluorescence can be detected using a stereoscopic camera [6]. ICG fluorescent angiography is generally performed intraoperatively before or after harvesting the flap to mark the area of likely flap survival. ICG fluorescent angiography was used to confirm a dynamic blood flow and provide a final confirmation of the perioperative posture of the perforators. This technique is also useful for determining which perforating branch is dominant [3].

The application of ICG, which has been used since the 1960s as a dye, has made ICG angiography safer owing to the superior pharmacokinetic as well as physiological and spectral properties of this substance, which allow for different clinical applications [7,8].

Detection techniques with P-CTA and magnetic resonance angiography are expensive and require radiation exposure. A combined examination is economically superior because ADS is free and ICG fluorescent angiography only costs the price of the ICG dye [9].

**Figure 6:**
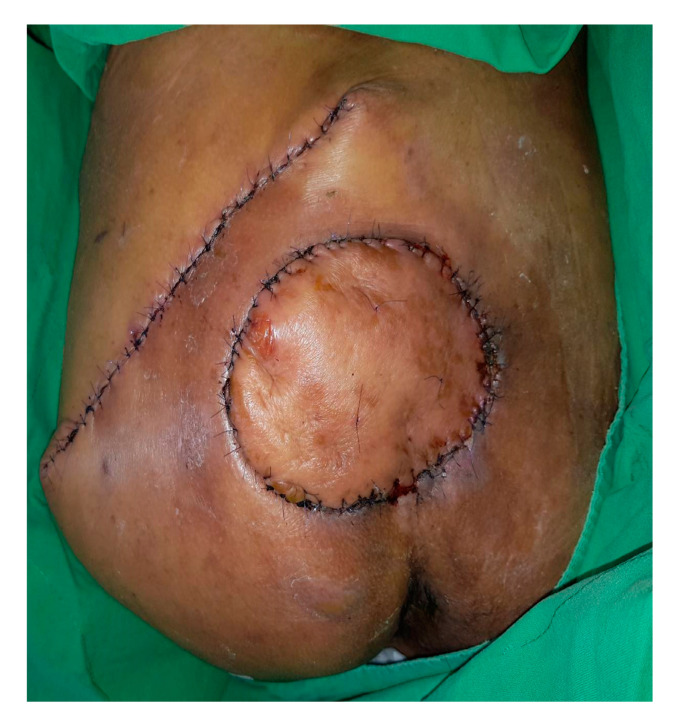
Post-operation day 10, the surgical wound is in the process of healing well.

In our report, the combination of ADS and ICGFA (Group 2) could detect more perforators than the ADS-only group (Group 1). The mean operative time was longer in group 2. However, this result is more effective when using ICGFA. The total number of hospital days was also reduced (Figure 6).

A key limitation of the present study is its retrospective character and small sample size. Despite these limitations and the bias involved through the missing randomization, small sample size, and surgeon’s preference of technique, we report the largest patient sample for the use of ADS and ICGFA for pressure injury reconstruction.

## 5. Conclusions

The combination of the detection of SGAP by ICGFA and by handheld ADS for the reconstruction of sacral pressure injury provides a more accurate method and provides the advantage of being radiation-free.

## Figures and Tables

**Table 1:** Retrospective reviewed data from first 8 patients.

Group 1	Sex/Age (Year)	BMI	ASA Status	Mobility Status	Albumin Level (g/dL)	Alcohol Use	Smoking History
1	F/75	19.2	3	DM/PAD	2.2	Denied	Denied
2	F/69	15.3	3	HTN	2.8	Denied	Denied
3	F/68	20.4	3	HTN	2.5	Denied	Denied
4	M/76	23.1	3	DM	2.6	Denied	Denied
5	F/82	17.5	3	CAD	2.9	Yes	Denied
6	M/72	16.8	3	HTN/CAD	2.3	Denied	Yes
7	F/87	19.4	3	DM	2.6	Denied	Denied
8	M/73	18.7	3	CAD	3.1	Denied	Yes

Ref: BMI: Body mass index; ASA status: American Society of Anesthesiologists status; DM: Diabetes mellitus; PAD: Peripheral artery occlusion disease; CAD: Coronary artery disease.

**Table 2:** Retrospective reviewed data from second 8 patients.

Group 2	Sex/Age (Year)	BMI	ASA Status	Mobility Status	Albumin Level (g/dL)	Alcohol Use	Smoking History
1	M/72	18.3	3	HTN	2.6	Denied	Denied
2	F/65	20.4	3	DM	2.9	Denied	Denied
3	F/78	16.4	3	CAD	2.1	Denied	Denied
4	M/70	18.7	3	DM/HTN	2.8	Yes	Yes
5	F/90	19.3	3	DM	2.9	Denied	Denied
6	M/82	16.9	3	HTN	2.5	Yes	Yes
7	F/69	17.2	3	CAD	3.1	Denied	Denied
8	M/72	16.2	3	CAD	2.3	Denied	Denied

Ref: BMI: Body mass index; ASA status: American Society of Anesthesiologists status; DM: Diabetes mellitus; PAD: Peripheral artery occlusion disease; CAD: Coronary artery disease.

**Table 3:** Group 1.

Patient	Sex/Age (Year)	Cause of Sacral Defect	Flap Size (cm^2^)	Perforator Number	Surgical Time (Mins)	Outcome	Hospital Days
1	F/75	Dementia/Bed-ridden	92	1	90	Good	15
2	F/69	Dementia/Bed-ridden	72	1	88	Good	17
3	F/68	Dementia/Bed-ridden	108	1	102	Good	14
4	M/76	Parkinson’s disease/Bed-ridden	49	2	96	Good	18
5	F/82	Stroke	84	1	80	Good	15
6	M/72	Stroke	62	1	110	Good	19
7	F/87	Stroke	90	1	72	Wound dehiscence	16
8	M/73	Spinal cord injury	72	1	90	Good	14

**Table 4:** Group 2 (ADS and ICGFA).

Patient	Sex/Age (Year)	Cause of Sacral Defect	Flap Size	Perforator Number ADS	Perforator Number ICGFA	Surgical Time	Outcome	Hospital Days
1	M/72	Dementia/Bed-ridden	90	2	2	100	Good	12
2	F/65	Dementia/Bed-ridden	68	1	2	92	Good	14
3	F/78	Parkinson’s disease/Bed-ridden	54	1	2	102	Wound dehiscence	12
4	M/70	Parkinson’s disease/Bed-ridden	81	2	2	112	Good	11
5	F/90	Parkinson’s disease/Bed-ridden	80	1	2	124	Good	13
6	M/82	Stroke	63	2	3	95	Good	15
7	F/69	Stroke	45	2	2	126	Good	14
8	M/72	Stroke	72	1	2	111	Good	13

## Data Availability

The data presented in this study are available in this article.
